# Clinical and Genetic Analysis of Costa Rican Patients With Parkinson's Disease

**DOI:** 10.3389/fneur.2021.656342

**Published:** 2021-08-04

**Authors:** Gabriel Torrealba-Acosta, Eric Yu, Tanya Lobo-Prada, Javier Ruíz-Martínez, Ana Gorostidi-Pagola, Ziv Gan-Or, Kenneth Carazo-Céspedes, Jean-François Trempe, Ignacio F. Mata, Jaime Fornaguera-Trías

**Affiliations:** ^1^Department of Neurology and Neurosurgery, Baylor College of Medicine, Houston, TX, United States; ^2^Neurosciences Research Center, Universidad de Costa Rica, San José, Costa Rica; ^3^Montreal Neurological Institute and Hospital, McGill University, Montreal, QC, Canada; ^4^Department of Human Genetics, McGill University, Montreal, QC, Canada; ^5^Department of Biochemistry, Medicine School, Universidad de Costa Rica, San José, Costa Rica; ^6^Group of Neurodegenerative Diseases, Biodonostia Health Research Institute, San Sebastian, Spain; ^7^CIBERNED, Centro de Investigación Biomédica en Red sobre Enfermedades Neurodegenerativas, Madrid, Spain; ^8^Movement Disorders Unit, Neurology Department, Donostialdea Integrated Health Organisation, Osakidetza Basque Health Service, San Sebastian, Spain; ^9^Genomic Platform, Biodonostia Health Research Institute, San Sebastian, Spain; ^10^Department of Neurology and Neurosurgery, McGill University, Montreal, QC, Canada; ^11^Department of Neurology, Hospital San Juan de Dios, Caja Costarricense de Seguro Social, San José, Costa Rica; ^12^Department of Pharmacology and Therapeutics and Centre de Recherche en Biologie Structurale, McGill University, Montreal, QC, Canada; ^13^Cleveland Clinic Foundation, Genomic Medicine, Lerner Research Institute, Cleveland, OH, United States

**Keywords:** Parkinson's disease, genotype, phenotype, Costa Rica, Latin America

## Abstract

**Background:** Most research in genomics of Parkinson's disease (PD) has been done in subjects of European ancestry, leading to sampling bias and leaving Latin American populations underrepresented. We sought to clinically characterize PD patients of Costa Rican origin and to sequence familial PD and atypical parkinsonism-associated genes in cases and controls.

**Methods:** We enrolled 118 PD patients with 97 unrelated controls. Collected information included demographics, exposure to risk and protective factors, and motor and cognitive assessments. We sequenced coding and untranslated regions in familial PD and atypical parkinsonism-associated genes including *GBA, SNCA, VPS35, LRRK2, GCH1, PRKN, PINK1, DJ-1, VPS13C*, and *ATP13A2*.

**Results:** Mean age of PD probands was 62.12 ± 13.51 years; 57.6% were male. The frequency of risk and protective factors averaged ~45%. Physical activity significantly correlated with better motor performance despite years of disease. Increased years of education were significantly associated with better cognitive function, whereas hallucinations, falls, mood disorders, and coffee consumption correlated with worse cognitive performance. We did not identify an association between tested genes and PD or any damaging homozygous or compound heterozygous variants. Rare variants in *LRRK2* were nominally associated with PD; six were located between amino acids p.1620 and 1623 in the C-terminal-of-ROC (COR) domain of Lrrk2. Non-synonymous *GBA* variants (p.T369M, p.N370S, and p.L444P) were identified in three healthy individuals. One PD patient carried a pathogenic *GCH1* variant, p.K224R.

**Discussion:** This is the first study that describes sociodemographics, risk factors, clinical presentation, and genetics of Costa Rican patients with PD, adding information to genomics research in a Latino population.

## Introduction

Parkinson's disease (PD) is a complex and heterogeneous movement disorder caused by a progressive degeneration of dopaminergic neurons. Main clinical motor symptoms associated with PD include tremor, rigidity, bradykinesia, and postural imbalance (1). Years before motor symptoms are manifested, there can be prodromal non-motor key features that include rapid eye movement (REM) sleep disorders, anosmia, and constipation (2). Cognitive impairment involving dysexecutive dysfunction with deficits in planning (3), shifting and sharing of attention (4), and problem solving (5), together with visuospatial dysfunction (6), can be also present from early stages of the disease. PD pathophysiology involves environmental factors as well as genetic variance, which provide insight into its molecular pathogenesis. Among environmental factors that contribute to PD risk are pesticide and herbicide exposure, welding, and well water consumption. There are also protective factors such as smoking, coffee consumption, and performing physical activity that may reduce the risk of developing PD (7).

Since the description of PD-associated mutations in the *SNCA* (8), other genes have been linked to autosomal dominant (AD) forms of familial PD, including *LRRK2* and *VPS35*. In addition, there are clinically and genetically diverse early-onset (EO) autosomal-recessive (AR) forms of PD with associated genes like *PRKN, PINK1*, and *DJ-1* that exhibit phenotypes similar to idiopathic PD, while other associated genes such as *VPS13C* and *ATP13A2* combine atypical features of parkinsonism like dystonia and early cognitive impairment, along with a poor response to levodopa (9). Large-scale genome-wide association studies (GWASs) have identified 90 variants for PD risk across 78 genomic regions, confirming *SNCA* and *GBA* as the most important ones (10). Different *GBA* locus present as strong risk factors for PD in both homozygous and heterozygous state, displaying a phenotype similar to idiopathic PD, yet with a faster rate of progression of cognitive and motor decline (11).

Clinical characterization of PD in Latin American and Hispanic populations has been scarce (12). Likewise, there is a lack of diversity in genomics with an overrepresentation of European-derived individuals, leading to sampling bias and leaving large populations underrepresented (13). Few genetic trials have been conducted in PD individuals from Latin American populations. Studies looking at *LRRK2* mutations have shown that their frequency varies across geographic areas and ethnicity groups. For the G2019S mutation in the *LRRK2* gene, frequencies range from 0.2 to 0.4% in Peruvian cohorts (14, 15), up to 4% in Uruguayans (14) and 5.45% in an Argentinian series (16–19). Likewise, the R1441G and R1441H mutations in this same gene seem to be uncommon in Latin American populations (0.3–0.8%) (14, 18). The LARGE-PD, a research consortium established among several Latin American countries, has been collecting data for what is the largest PD cohort in the region, allowing for large-scale genotyping as well as performing GWAS in these cohorts (20–22). This initiative aimed to estimate the frequency of *LRRK2* mutations in the region and reported varying frequencies of the G2019S and R1441G/C mutations, which strongly correlated with the European admixture of the samples analyzed (15, 20).

*GBA* mutations have also been studied in few Latin American cohorts but mainly focused on most frequently reported mutations in other populations. The observed frequency of these mutations varies across regions ranging from 0.2% (p.N370S) to 0.7% (p.E326K) in Ecuadorians (23) and up to 5.5% (p.L444P) in Mexican Mestizo and Brazilian cohorts (23–27). Few studies have studied the entire *GBA* gene in Latin America, showing a frequency similar to those reported in individuals of European descent (4–5%), but lower than frequencies reported in Ashkenazi patients (20%) (28). Moreover, the overall frequency of *GBA* mutations seems to be consistently higher than *LRRK2* mutations across different geographic areas, suggesting that *GBA* could play a more important role in PD genetics for Latin American populations. Velez-Pardo et al. found a mutation that was specific for a Colombian cohort (p.K198E) and in a much higher frequency (9.9%) highlighting the need to sequence the whole *GBA* gene rather than focusing only on assessing commonly reported mutations (27).

In this study, we sought to clinically characterize PD patients of Costa Rican origin and to sequence familial PD and atypical parkinsonism-associated genes in Costa Rican PD cases and controls.

## Materials and Methods

### Study Subjects

We enrolled 118 consecutive unrelated PD patients (68 males, 50 females) with 97 unrelated controls (28 males, 69 females), matched according to age and gender whenever possible. Thirty-five patients (16.28%) reported having a relative (≤ 2°) with any sort of movement disorder; of those, 21 (9.77%) had a formal PD diagnosis. All subjects resided and were originated from Costa Rica and were recruited at the Movement Disorders Unit of the Department of Neurology, Hospital San Juan de Dios, Caja Costarricense de Seguro Social. All patients fulfilled Gelb criteria for the clinical diagnosis of PD, while controls had no signs or personal history of any neurodegenerative disease and were mainly the spouses of the PD cases. We preferred using Gelb criteria over the United Kingdom Parkinson's Disease Society Brain Bank (UKPDSBB) as it provided different clinical diagnostic levels of certainty (possible and probable) and it has shown to have similar positive and negative predictive values, as well as sensitivity and global accuracy when compared to UKPDSBB (29). Albeit both diagnostic criteria sets have low specificity and are mainly focused on motor features, UKPDSBB criteria further err by challenging PD diagnosis in the presence of genetic risk factors (30). Our last patient was enrolled by 2011, which is 4 years earlier than when the Movement Disorder Society (MDS) task force proposed the new clinical diagnostic criteria for PD (MDS-PD criteria) (31); therefore, we were not able to use those for clinical diagnosis of patients enrolled in our study.

We gathered information concerning work and educational status as well as history of exposure to risk and protective factors of PD. We further obtained detailed information on PD history, comorbidities, and antiparkinsonian treatments. Additionally, motor disability of the patients was evaluated by means of the Unified Parkinson's Disease Rating Scale (UPDRS), Hoehn & Yahr (H&Y), and Schwab & England (S&E) scales. Cognitive status was assessed using the Montreal Cognitive Assessment (MoCA) test.

### Genetic Analysis

Molecular inversion probes were used to sequence coding and untranslated regions in familial PD and atypical parkinsonism-associated genes including *GBA, SNCA, VPS35, LRRK2, GCH1, PRKN, PINK1, DJ-1, VPS13C*, and *ATP13A2* at McGill University with Illumina HiSeq 4000 as previously described (32). The full protocol can be found at https://github.com/gan-orlab/MIP_protocol. All sequences were aligned using Burrows-Wheeler Aligner (BWA) using the reference genome hg19 (33). Genome Alignment Tool Kit (GATK v3.8) was used to call variants and perform quality control and ANNOVAR was used to annotate each variant (34, 35). Exons 10 and 11 of *GBA* were sequenced using Sanger sequencing as previously described (36), and *GBA* variants in other exons were also validated using Sanger sequencing. We decided to focus on genes that are involved in typical PD, as our selected cohort is of typical PD (10, 37, 38).

### Quality Control

All samples and variants were filtered based on standard quality control process as previously reported (39). In brief, variants were separated into common and rare by minor allele frequency (MAF) in the cohort. Rare variants (MAF < 0.01) with a minimum depth of coverage of > 30× were included in the analysis, along with common variants (MAF ≥ 0.01) with >15× coverage. We have established that for common variants, we get reliable reads at 15×; however, to get reliable reads for rare variants, we need > 30×; otherwise, there are many false positives (40). Variant calls with a genotype frequency of <25% of the reads or genotype quality of <30 were excluded. Samples and variants with more than 10% missingness were also excluded.

### *In silico* Structural Analysis

The atomic coordinates of the human Lrrk2 C-terminal domain structure (a.a. 1327–2527) were downloaded from the Protein Data Bank (ID 6VP6). The figure was generated using PyMol v.2.4.0.

### Statistics

We used Stata® (version 14) for the statistical analysis of sociodemographic and clinical variables. Normally distributed variables are reported as mean with its standard deviation (SD), whereas continuous but non-normally distributed variables are reported as median with the 25th and 75th percentile values (interquartile range, IQR). Normally distributed variables were compared with paired or unpaired *t-*tests, while non-normally distributed variables were compared with Mann–Whitney *U*-test or Wilcoxon match-paired signed-rank test. Frequencies were compared with **χ**^2^ and Fisher's exact test. Tests were two-tailed, and significance was set at *p* < 0.05. We modeled through linear regression the association between demographic and clinical variables with the severity of the disease, as indexed by UPDRS and MoCA, as dependent variables in the models.

For genetic analysis, common and rare variants were analyzed separately. Association of common variants was tested using logistic regression adjusted for age and sex in PLINK v1.9. For rare variants' analysis, we examined the burden of rare variants in each gene using optimized sequence Kernel association test (SKAT-O) adjusted for age and sex (41). Rare variants were separated into different categories based on their potential pathogenicity to examine specific enrichment in different variant subgroups as described previously (40): (1) variants with Combined Annotation Dependent Depletion (CADD) score of ≥12.37 (representing the top 2% of potentially deleterious variants) (42); (2) regulatory variants predicted by ENCODE (43); (3) potentially functional variants including all non-synonymous variants, stop gain/loss variants, frameshift variants, and intronic splicing variants located within two base pairs of exon–intron junctions; (4) loss-of-function variants, which includes stop gain/loss, frameshift, and splicing variants; and (5) only non-synonymous variants. Bonferroni correction for multiple comparisons was applied as necessary.

This study was approved by the Ethics Committee of Hospital San Juan de Dios, Caja Costarricense de Seguro Social (CLOBI-HSJD #014-2015) and the University of Costa Rica (837-B5-304). Written informed consent was obtained from all participants.

## Results

### Sociodemographic and Clinical Variables

At enrollment, PD probands had a mean age of 62.12 ± 13.51 years (range 25–86), and the mean age at onset was 54.62 ± 13.54 (range 16–83) years. Male PD patients comprised 57.63% of the sample. Despite the fact that a significantly larger proportion of the male PD patients reported current or previous jobs involving agricultural activities (19.40% male, 2.08% female; *p* = 0.01), the mean number of years of education of these men was significantly higher than women (10.74 ± 3.81 vs. 8.86 ± 4.01; *p* = 0.03). Table 1 details subjects' baseline characteristics along with the frequency of exposure to main risk and protective factors for PD. Most of the risk and protective factors were more prevalent in men. Tables 1, 2 detail the frequency of clinical manifestations as well as the standardized scale scores reported for PD cases. The most frequent initial symptoms included resting tremor (71.30%), rigidity (24.07%), and pain (10.19%). Most of the patients had asymmetric onset (94.12%) and a good response to levodopa (89.11%). Other frequently reported motor features comprised dystonia (46.08%), falls (39.22%), and dysphagia (36.27%). Common non-motor manifestations such as hyposmia, sleep disorders and depressive/anxious mood were seen in more than 50% of the cases. Overall median score of UPDRS “ON” was 36 (22–60), most of our patients were graded in the “2.5” and “3” categories of the H&Y scale with a median for S&E score of 80% (80–90%). The median value for the MoCA test was 22 (17–25). There were no statistically significant differences between sex, regarding these scores.

**Table 1:** Baseline characteristics with frequency of risk and protective factors for PD in study subjects, with sex comparison.

	**Men**	**Women**	**Total**	***p***
	**96 (44.65%)**	**119 (55.35%)**	**(*n* = 215)**	
Condition				**<0.001**
Cases	68/118 (57.63%)	50/118 (42.37%)	118/215 (54.88%)	
Controls	28/97 (28.87%)	69/97 (71.13%)	97/215 (45.12%)	
Age of onset (mean ± SD)	54.74 ± 12.03	54.46 ± 15.49	54.62 ± 13.54	0.91
Age of recruitment (mean ± SD)	62.75 ± 12.17	61.26 ± 15.22	62.12 ± 13.51	0.1
Years of education^†^ (mean ± SD)	10.74 ± 3.81	8.86 ± 4.01	9.94 ± 3.98	**0.03**
Agricultural activities^†^, *n* (%)	13/67 (19.40%)	1/48 (2.08%)	14/115 (12.17%)	**0.01**
Risk factors^†^, *n* (%)				
Pesticides	24/67 (35.82%)	10/48 (20.83%)	34/115 (29.57%)	0.1
Herbicides	26/67 (38.81%)	9/48 (18.75%)	35/115 (30.43%)	**0.03**
Welding	22/65 (33.85%)	3/48 (6.25%)	25/113 (22.12%)	**<0.001**
Heavy metals	11/64 (17.19%)	2/48 (4.17%)	13/112 (11.61%)	**0.04**
Non-potable water	29/66 (43.94%)	19/48 (39.58%)	48/114 (42.11%)	0.7
Cardiovascular	41/58 (70.69%)	32/43 (74.42%)	73/101 (72.28%)	0.82
Years of exposure, median (IQR)	8 (1–20)	5 (1–19)	8 (1–20)	0.36
Protective factors^†^, *n* (%)				
Smoking	36/67 (53.73%)	9/48 (18.75%)	45/115 (39.13%)	**<0.001**
Coffee	61/65 (93.85%)	44/48 (91.67%)	105/113 (92.92%)	0.72
Alcohol	43/67 (64.18%)	9/48 (18.75%)	52/115 (45.22%)	**<0.001**
Physical activity	47/67 (70.15%)	17/48 (35.42%)	64/115 (55.65%)	**<0.001**
UPDRS “on” median (IQR)				
I	2 (0.5–4)	2 (0–5)	2 (0–5)	0.98
II	9 (3–17)	8 (3–14)	9 (3–16)	0.57
III	23 (10–35)	26 (14–37)	23 (12–36)	0.57
Total	35 (21–59)	36.5 (23–60)	36 (22–60)	0.97
Hoehn and Yahr				
1	5/59 (8.47%)	9/43 (20.93%)	14/102 (13.73%)	0.36
1.5	7/59 (11.86%)	6/43 (13.95%)	13/102 (12.75%)	
2	12/59 (20.34%)	5/43 (11.63%)	17/102 (16.67%)	
2.5	14/59 (23.73%)	9/43 (20.93%)	23/102 (22.55%)	
3	17/59 (28.81%)	8/43 (18.60%)	25/102 (24.51%)	
4	3/59 (5.08%)	4/43 (9.30%)	7/102 (6.86%)	
5	1/59 (1.69%)	2/43 (4.65%)	3/102 (2.94%)	
Schwab and England, median (IQR)	80 (80–90)	90 (80–90)	80 (80–90)	0.33
MoCA test, median (Q1–Q3)	22.5 (18–25.5)	22 (14–25)	22 (17–25)	0.38

† *Information available only for patients and does not include controls*.

*IQR, interquartile range (Q1–Q3); MoCA, Montreal Cognitive Assessment; PD, Parkinson's disease; SD, standard deviation; UPDRS, Unified Parkinson's Disease Rating Scale. The significance was set at p < 0.05 were indicated in bold*.

**Table 2:** Clinical manifestations of PD cases, with sex comparison.

	**Men 68 (57.63%)**	**Women 50 (42.37%)**	**Total**	***p*** (***n***= 118)
**Initial symptoms**, ***n*** **(%)**				
Resting tremor	43/63 (68.25%)	34/45 (75.56%)	77/108 (71.30%)	0.52
Rigidity	17/63 (26.98%)	9/45 (20.00%)	26/108 (24.07%)	0.5
Postural instability	3/63 (4.76%)	1/45 (2.22%)	4/108 (3.70%)	0.64
Bradykinesia	3/63 (4.76%)	1/45 (2.22%)	4/108 (3.70%)	0.64
Pain	3/63 (4.76%)	8/45 (17.78%)	11/108 (10.19%)	**0.03**
**Symptoms**, ***n*** **(%)**				
Resting tremor	51/59 (86.44%)	40/43 (93.02%)	91/102 (89.22%)	0.35
Bradykinesia	55/59 (93.22%)	39/43 (90.70%)	94/102 (92.16%)	0.72
Rigidity	50/59 (84.75%)	36/43 (83.72%)	86/102 (84.31%)	0.89
Asymmetry	57/59 (96.61%)	39/43 (90.70%)	96/102 (94.12%)	0.24
Levodopa response	55/59 (93.22%)	35/42 (83.33%)	90/101 (89.11%)	0.19
Hallucinations	14/59 (23.73%)	8/43 (18.60%)	22/102 (21.57%)	0.63
Orthostatism	9/59 (15.25%)	12/43 (27.91%)	21/102 (20.59%)	0.14
Falls	23/59 (38.98%)	17/43 (39.53%)	40/102 (39.22%)	0.96
Syncope	1/59 (1.69%)	2/43 (4.65%)	3/102 (2.94%)	0.57
Dystonia	28/59 (47.46%)	19/43 (44.19%)	47/102 (46.08%)	0.84
Dysphagia	20/59 (33.90%)	17/43 (39.53%)	37/102 (36.27%)	0.68
Hyposmia	34/63 (53.97%)	23/47 (48.94%)	57/110 (51.82%)	0.7
Constipation	23/52 (44.23%)	13/41 (31.71%)	36/93 (38.71%)	0.29
Urinary symptoms	7/52 (13.46%)	5/41 (12.20%)	12/93 (12.90%)	0.86
Sleep disorders	52/65 (80.0%)	37/52 (71.2%)	89/116 (76.7%)	0.26
Insomnia	22/52 (42.31%)	16/37 (43.24%)	38/89 (42.70%)	0.93
Vivid dreams	26/52 (50.00%)	13/37 (35.14%)	39/89 (43.82%)	0.2
Mood disorders (depression or anxiety)	40/63 (63.49%)	29/47 (61.70%)	69/110 (62.73%)	0.85
Disease duration (years), median (IQR)	5 (3–10)	5 (3–7)	5 (3–9)	0.18

*IQR, interquartile range (Q1–Q3). The significance was set at p < 0.05 were indicated in bold*.

We were able to establish through multivariate linear regression modeling that an increased disease duration along with the presence of orthostasis, dysphagia, and mood disorders significantly correlated with increased scores in total ON UPDRS. Furthermore, we found an interaction between performing regular physical activity and duration of disease, where despite having increased years of evolution, patients that performed regular physical activity still scored less in the total ON UPDRS (see Supplementary Figure 1). Additionally, lower scores in MoCA testing significantly correlated with increased age, coffee consumption, and the presence of hallucinations, falls, and mood disorders (depression/anxiety), whereas increased years of education correlated with better MoCA scores (see Supplementary Figure 2).

### Quality of Coverage and Identified Variants

The average coverage of the 10 genes analyzed in this study was >588× for all genes. The coverage per gene and the percentage of nucleotides covered at >15× and >30× for each gene are detailed in Supplementary Table 1. There were no differences in the coverage across the samples (patients and controls). Overall, after quality control, we identified 163 rare variants (Supplementary Table 2) and 158 common variants (Supplementary Table 3) across all genes and all samples that were included in the analysis. Specific protein and DNA changes are listed in Supplementary Tables 4, 5 for rare and common exonic variants, respectively.

### Rare and Common Variants in PD and Parkinsonism-Related Genes

Burden and SKAT-O analyses did not identify an association of any of the tested genes and PD (Table 3) after correction for multiple comparisons, as expected given the small sample size. We also did not identify any PD patients with potentially damaging homozygous or compound heterozygous variants in any of these genes. Rare variants in *LRRK2* were nominally associated with PD, and 11 (9.2%) patients carried a rare non-synonymous variant, compared to four (4.1%) among the controls. Interestingly, six of these rare non-synonymous variants, all located between amino acids p.1620 and 1623 in the C-terminal-of-ROC (COR) domain of Lrrk2, were found in six patients and none in controls (Table 4).

**Table 3:** Burden and SKAT-O analyses with no significant association found of any of the tested genes and PD, after Bonferroni correction for multiple comparisons.

	**All**		**CADD**		**Encode**		**Funct**		**LOF**		**NS**	
	**Burden**	**SKATO**	**Burden**	**SKATO**	**Burden**	**SKATO**	**Burden**	**SKATO**	**Burden**	**SKATO**	**Burden**	**SKATO**
*LRRK2*	0.017	0.030	0.127	0.265	0.400	0.682	0.061	0.123	NA	NA	0.087	0.160
*VPS35*	0.341	0.341	NA	NA	NA	NA	NA	NA	NA	NA	NA	NA
*SNCA*	0.045	0.101	NA	NA	0.347	0.560	0.347	0.560	NA	NA	NA	NA
*GCH1*	0.764	0.880	0.209	0.209	0.722	0.722	0.753	0.624	NA	NA	0.209	0.209
*PRKN*	0.839	0.967	0.874	0.900	NA	NA	0.874	0.900	NA	NA	0.874	0.900
*PINK1*	0.722	0.860	0.200	0.355	NA	NA	0.352	0.582	NA	NA	0.352	0.582
*PARK7*	0.586	0.779	0.779	0.779	0.812	0.897	0.546	0.778	NA	NA	0.779	0.779
*VPS13C*	0.274	0.406	0.563	0.808	0.095	0.095	0.195	0.349	0.332	0.767	0.829	0.952
*ATP13A2*	0.054	0.137	0.791	0.397	NA	NA	0.791	0.397	NA	NA	0.791	0.397

*The numbers represent the uncorrected p-values of the tests. Burden, burden test; SKATO, optimized sequence Kernel association test; CADD, Combined Annotation Dependent Depletion (CADD) score of ≥ 12.37 (representing the top 2% of potentially deleterious variants); Encode, regulatory variants predicted by ENCODE; Funct, Potentially functional variants including all non-synonymous variants, stop gain/loss variants, frameshift variants, and intronic splicing variants located within two base pairs of exon–intron junctions; LOF, loss-of-function variants, which include stop gain/loss, frameshift, and splicing variants; NS, only non-synonymous variants; NA, not applicable—not enough variants for analysis*.

**Table 4:** Rare variants in *LRRK2* present in patients and controls.

**SNP location/rs number** **and nucleotide change**	**Detailed annotation of the variant (DA)**	**Status**	**Family history**
12:40709172:T:C	intronic:LRRK2:NM_198578	Control	
12:40709180:T:C	intronic:LRRK2:NM_198578	Affected	
12:40709181:T:C	intronic:LRRK2:NM_198578	Affected	
rs760912433:C:T	exonic:non-synonymous_SNV:LRRK2:NM_198578:exon34:c.C4856T:p.P1619L	Control	
12:40713821:A:G^†^	exonic:non-synonymous_SNV:LRRK2:NM_198578:exon34:c.A4859G:p.K1620R	Affected	
12:40713824:A:G^†^	exonic:non-synonymous_SNV:LRRK2:NM_198578:exon34:c.A4862G:p.H1621R	Affected	Mother: • Epilepsy (type unknown) diagnosed in early adulthood. • Dementia associated to rigidity, diagnosed at 67 years old, death at 69 years old • Retinitis pigmentosa
rs765275134:C:A^†^	exonic:non-synonymous_SNV:LRRK2:NM_198578:exon34:c.C4863A:p.H1621Q	Affected	
12:40713826:C:A^†^	exonic:non-synonymous_SNV:LRRK2:NM_198578:exon34:c.C4864A:p.P1622T	Affected	Father: • Dementia (type unknown), not associated to hallucinations or motor symptoms. Diagnosed at 74 years old, death at 84 years old.
rs751492506:C:T^†^	exonic:non-synonymous_SNV:LRRK2:NM_198578:exon34:c.C4865T:p.P1622L	Affected	Two sisters (from both parents): • Parkinson's disease diagnosed at ages 30 and 20 years old. Maternal aunt: • Bilateral hand tremor (described as intention tremor, does not have a definitive diagnosis). Maternal great-grandfather: • Bilateral hand tremor (type unknown).
12:40713828:T:C	exonic:synonymous_SNV:LRRK2:NM_198578:exon34:c.T4866C:p.P1622P	Control	
12:40713829:A:G^†^	exonic:non-synonymous_SNV:LRRK2:NM_198578:exon34:c.A4867G:p.K1623E	Affected	Mother: • Cirrhosis (not associated to neurologic symptoms). Maternal uncle: • Parkinson's disease (diagnosed at 50 years old). Maternal grandfather: • Tremor in both hands (type unknown).
rs73097447:A:C	intronic:LRRK2:NM_198578	Affected	
12:40716092:G:T	intronic:LRRK2:NM_198578	Affected and Control	

*Six of these rare non-synonymous variants (^†^, all located between amino acids p.1620 and 1623 in the COR domain of LRRK2, were found only in patients and not in controls*.

Non-synonymous *GBA* variants were identified in three individuals: p.T369M was identified in a male patient with age at onset of 48 years, p.N370S was identified in a healthy female individual recruited at the age of 78 years, and p.L444P was identified in a healthy female individual recruited at the age of 64. While we cannot rule out that these healthy individuals will develop PD in the future, it is unlikely that *GBA* variants have a major role in PD among Costa Rican patients. One PD patient carried a pathogenic *GCH1* variant, p.K224R, further emphasizing the role of this gene in PD.

In the analysis of common variants, none of the variants was associated with PD after correction for multiple comparisons (Supplementary Table 3), which set the corrected *p*-value for statistical significance at *p* < 0.00031. One non-synonymous variant in *LRRK2*, p.I723V, was found with allele frequency of 0.01 in patients and 0.09 in controls (OR = 0.11, 95% CI = 0.02–0.52, *p* = 0.005), yet this difference was not statistically significant after correction for multiple comparisons.

## Discussion

### Clinical Features

PD prevalence has been increasing over time with a global age-standardized prevalence rate increase of 21.7% from the years 1990 to 2016 (44). Furthermore, PD prevalence seems to be lower in Eastern compared to Western countries (45). Few studies have explored the prevalence of PD in Latin America providing values that are similar either to other developing countries (46) or to European cohorts (47, 48). PD also becomes more common with advancing age (44, 45). Our sample average age of PD at onset and at diagnosis was lower when compared to other cohorts (49–51), although it could suggest that PD presents earlier in Costa Rica, and more epidemiological studies are needed as it could also be related to recruitment bias.

The majority of our patients fulfilled Group A Gelb criteria while up to 60% also reported at least one of Group B symptoms, the most frequent being dystonia, falls, and dysphagia. The median for years of evolution of the disease for both men and women was 5; thus, we would expect to find Group B criteria in these patients along with the evolution of the disease. Few studies have explored ethnic variations in motor symptoms of PD, suggesting increased atypical features in Black and South Asian PD patients (52, 53); however, there is not enough evidence available along with a lack of standardized methodology to determine motor subtypes across studies and to further establish ethnic patterns of motor features (12). Common non-motor manifestations such as hyposmia, sleep disorders, and depressive/anxious mood were seen in more than half of our PD cases. Regardless of ethnicity, non-motor features are commonly present in PD with subtle differences described. Gastrointestinal non-motor features along with depression seem to be high in East Asian cohorts (54, 55). Likewise, Latino populations, such as Mexican (56), Peruvian (57), and ours, also reported high frequency of mood disorders including depression and anxiety, when compared to studies from UK and USA (58, 59). We also observed in our sample a frequency of sleep disorders and hyposmia that is higher than those reported in other cohorts (12).

Overall, our patients had a low education, which has been previously associated with a higher hazard of incident parkinsonism (60). A reduced education has also been suggested as a risk factor for cognitive impairment in PD (61). A history of non-potable water consumption along with exposure to pesticides and herbicides was reported in up to 40% of our patients. This type of exposure agrees with a mostly rural origin and the fact that 12.2% of the subjects reported involvement in agricultural activities as a main income source. We did not assess the frequency of protective and risk factors in the control group; hence, we are not able to establish any comparison with PD cases. Previous exposure to pesticides and herbicides is associated with the development of PD (62); yet, the identification of a given specific agent and the exact timing and dosing of exposure are almost impossible to establish through observational studies (63, 64). Nonetheless, key work detailing specific mechanisms that render patients vulnerable to pesticide-induced injury has been elegantly shown in animal models, further establishing biologic and toxicological pathways for specific chemicals to potentially cause PD (65). A similar situation is present regarding the exposure to welding and heavy metals. Manganese (66), copper, iron (67), and mercury (68) have been proposed as possible agents associated with the development of PD. In this study, 22.1 and 11.6% of the patients reported frequent exposure to welding and other heavy metals, respectively; however, the exact timing and dosing of exposure was not possible to assess.

Other literature has underscored the presence of protective factors for PD development, among which the most notable and with the strongest evidence include tobacco (69) and coffee consumption (7, 70–73). For both protective factors, there is also a dosing effect described, where the protective effect increases along with an increasing exposure (74, 75). Paradoxically, over 90% of our PD cases had been exposed to a protective factor in the past, most of them having a regular coffee intake (two to three cups per day for over 15 years), and yet they all developed PD.

Performing regular physical activity correlated with lower ON UPDRS scores in spite of increasing age. Physical activity has been established as a possible protective factor for incident Parkinsonism (76); our data would suggest that physical activity could determine reduced severity of disease, specifically concerning motor features. Although exercise has not been proven to slow the progression of akinesia, rigidity, and gait disturbances, it promotes a feeling of physical and mental well-being, and at the same time, it can alleviate rigidity-related pain and improve patients' motor (77) and non-motor symptoms (78).

Increasing age, coffee consumption, hallucinations, falls, and mood disorders along with reduced years of education significantly correlated with worse MoCA scores. Older age and duration of PD are determinant risk factors for incidence of dementia in PD (79). Furthermore, hallucinations have been established as risk factors for cognitive impairment (79, 80) along with gait disturbances (manifested by falls) (81) and depression (82). Reduced education years also have been proposed as a risk factor for cognitive impairment in patients with PD (61). Poor global cognition has been previously associated with a higher risk of incident parkinsonism (60). Coffee consumption has been suggested to reduce risk of dementia (83) with a dosing effect (84, 85); however, there have been inconsistent findings regarding the effects of coffee consumption on specific cognitive domains. It has been suggested to be in association with improved executive performance but smaller hippocampal volume and worse memory function (86); nonetheless, this association is not sustained when cognition is analyzed longitudinally. Other literature suggested that coffee might be slightly beneficial on memory without a dose–response relationship (87). Recent large-scale genetic analysis using mendelian randomization did not find any evidence supporting any beneficial or adverse long-term effect of coffee consumption on global cognition or memory function (88) or AD incidence (89). To our knowledge, there is no literature evaluating the effect on cognition of coffee consumption, specifically for PD patients. Our findings suggest a possible deleterious effect that should be further explored in this population.

### Genetic Assessment

After sequence coding familial PD and atypical parkinsonism-associated genes including *GBA, SNCA, VPS35, LRRK2, GCH1, PRKN, PINK1, DJ-1, VPS13C*, and *ATP13A2* and correcting for multiple comparisons, burden and SKAT-O analyses did not show an association of any of the tested genes and PD. We also did not identify any homozygous or compound heterozygous pathogenic variants in any of these genes.

Non-synonymous *GBA* variants were identified in three individuals including one patient and two unaffected controls. While we cannot rule out that these healthy individuals will develop PD in the future, it is unlikely that *GBA* variants have a major role in PD among Costa Rican patients especially when compared to other European and Ashkenazi Jewish populations where we find that 8–20% of the patients harbor *GBA* variants (28).

Finally, one PD patient carried a pathogenic variant, p.K224R, in the *GCH1* gene. *GCH1* encodes for GTP cyclohydrolase 1, which is a key enzyme for dopamine production in nigrostriatal neurons. Loss-of-function mutations such as p.K224R have been shown to cause Dopa-responsive dystonia (DRD); however, variants in this gene have also been implicated in PD, perhaps through regulation of *GCH1* expression (90, 91). It has been suggested that late-onset DRD might present clinically with parkinsonism, or alternatively, pathogenic *GCH1* mutations may predispose to both diseases and carriers will develop any or both depending on other genetic or environmental factors (92). Our patient did not present clinical features suggestive of DRD and did not have any family history of PD.

Rare variants in *LRRK2* were nominally associated with PD, observed only in affected individuals; six of these rare non-synonymous variants were located between amino acids p.1620 and 1623 in the COR domain of Lrrk2. *LRRK2* encodes a multiple domain protein that includes a Roc-COR tandem domain, a tyrosine kinase-like protein kinase domain, and at least four repeat domains located within the N-terminal and C-terminal regions. The Roc-COR domain classifies the Lrrk2 protein as part of the ROCO superfamily of Ras-like G proteins (93). Mutations in *LRRK2* are the most common cause of late-onset hereditary PD. Most frequently reported disease-causing mutations are located in the kinase domain (i.e., G2019S), increasing kinase activity, and in the Roc-COR tandem domain (i.e., R1441C/G and Y1699C), impairing its GTPase function. Alterations of both kinase and GTPase activity may mediate neurodegeneration in these forms of PD (94). Of the six patients found to have non-synonymous variants in the COR domain, two had first-degree relatives with dementia, one had a second-degree relative with PD, and one had two sisters with PD diagnosed at a very young age (20 and 30 years old) (see Table 4).

Methodological issues, such as size and composition of the sample (i.e., number of familial and sporadic cases), might explain the variations seen in the frequency of *LRRK2* mutations in case series from similar countries. However, there is a clear difference established among geographical regions, where North African Arabs (95), Ashkenazy-Jews (96) and certain Europeans cohorts (97–99) might report a higher prevalence than Latin American and Asian populations for these mutations (15, 100, 101).

### Structural Analysis of *LRRK2* Pathogenic Mutations

**Figure 1:**
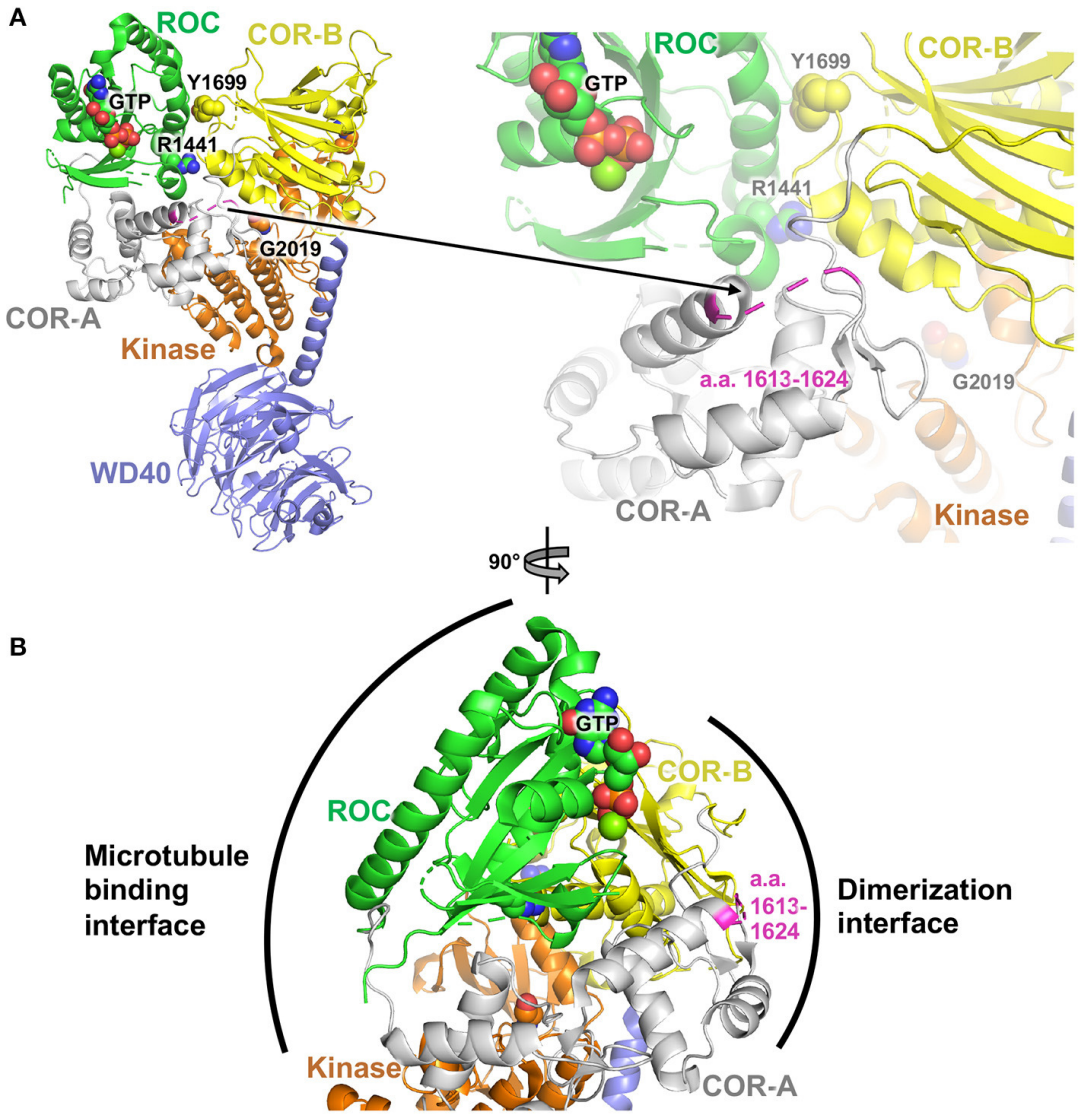
Structural analysis of PD variants in LRRK2. **(A)** Cryoelectron microscopy structure of Lrrk2 C-terminal domains, PDB code 6vp6 (102). Parkinson-linked missense mutation sites R1441, Y1699, and G2019 are shown as spheres. The loop spanning a.a. 1612–1624 in the COR-A domain is shown in magenta. **(B)** Rotated view (90°) of the structure in **(A)**, showing the proposed dimerization and microtubule binding interfaces, based on integrative modeling of Lrrk2 filaments bound to microtubules (102).

The non-synonymous missense mutations described here are all found in the COR domain of Lrrk2. To gain insight into how these mutations may affect the function of Lrrk2, we investigated their locations in the structure of Lrrk2. The high-resolution cryoelectron microscopy (cryoEM) structure of the C-terminal domains of Lrrk2 in different states have recently been reported and shed light on how allosteric interactions between different domains regulate microtubule interactions (102). The structure notably shows interactions between the ROC GTPase domain and the COR-B domain, notably involving the pathogenic mutation sites p.Arg1441 and p.Tyr1699 (Figure 1A). These interdomain interactions enable the kinase activity to be regulated by GTP binding to the ROC domain. The mutations described here, found in the segment a.a. 1619–1623, are all located in a loop of the COR-A domain. This loop, which spans a.a. 1613–1624, is disordered in the cryoEM structure, and thus, no atomic resolution model is available for that segment (Figure 1A). It is therefore not possible to gain detailed insights into the effect of each individual missense mutation.

However, integrative modeling, based on cryoelectron tomography (cryoET) data collected from *in situ* and *in vitro*-reconstituted Lrrk2 filaments bound to microtubules, shows how the different domains of Lrrk2 dimerize and associate with microtubules (102, 103). Dimerization is mediated via two sites through reciprocal interactions: one involving WD40–WD40 interactions and another one involving COR–COR interactions. These interactions enable Lrrk2 C-terminal domains to form extended oligomeric filaments that form a helix around the microtubule. Of particular interest here, the COR–COR dimerization interface involves both the COR-A and COR-B domains, with the loop containing a.a. 1613–1624 at the center of this interface (Figure 1B). Mutations in this loop may thus affect dimerization. Given that the kinase activity and conformation affect the ability of Lrrk2 to dimerize through the COR domain *via* allosteric interactions, it is possible that mutations in the COR-A loop in turn affect the kinase activity. Further experiments would be required to determine how the mutations described here affect the kinase, dimerization, and microtubule-binding activity of Lrrk2.

## Limitations

Genome analysis from Mestizo populations in Latin America has previously shown in Costa Rica a European, Native American, and African admixture of 66.7, 28.7, and 4.6%, respectively (104). Therefore, we would have expected to observe a higher frequency of mutations, similar to other European series reported. However, our sample size is small and is more representative of the metropolitan area where most of the patients were recruited, thus warranting in the future a more comprehensive study involving a wider and more representative population of the whole country, particularly including more patients from the non-metropolitan and coastal zones. Moreover, the purpose of our study was to serve as an exploratory analysis in this population, which had not been studied before; likewise, we opted to cover as many genes as possible. We are aware that the sample size is limited, yet underrepresented populations with limited funding and resources that struggle to achieve large sample sizes should be studied and reported as well.

We did not gather information concerning protective and risk factors for subjects in the control group, therefore, we were not able to compare and discuss the frequency of these factors between cases and controls.

## Conclusions

This is the first study that reports on sociodemographics, risk factors, clinical presentation, and genetics of Costa Rican patients with PD. We observed a high frequency of exposure to both risk factors (pesticides, herbicides, non-potable water, and low education) and protective factors (tobacco and coffee intake). Regular physical activity significantly correlated with better UPDRS scores despite years of evolution of the disease. Increased years of education were significantly associated with better MoCA test scores, whereas the presence of hallucinations, falls, and mood disorders correlated with a worse performance in the MoCA test. Interestingly, coffee consumption also correlated significantly with worse MoCA test scoring.

We did not find an association between any of the tested familial PD and atypical parkinsonism-associated genes, including *GBA, SNCA, VPS35, LRRK2, GCH1, PRKN, PINK1, DJ-1, VPS13C*, and *ATP13A2*, and PD. We also did not identify any homozygous or compound heterozygous pathogenic variants in any of these genes. Rare variants in *LRRK2* were nominally associated with PD, with six of these rare non-synonymous variants all located in the COR domain of *LRRK2*. One PD patient carried a pathogenic *GCH1* variant, p.K224R, further emphasizing the role of this gene in PD.

## Data Availability Statement

The data presented in the study are deposited in the NIH-dbGAP repository, accession number phs002495.v1.p1 (http://www.ncbi.nlm.nih.gov/projects/gap/cgi-bin/study.cgi?study_id=phs002495.v1.p1).

## Ethics Statement

The studies involving human participants were reviewed and approved by the Ethics Committee of the University of Costa Rica (VI-3668-2014 and final report 837-B5-302). The patients/participants provided their written informed consent to participate in this study.

## Author Contributions

GT-A and EY conceptualized the report and made substantial contributions to the design, drafting, and revision of the work. J-FT performed *in silico* structural analysis and contributed to the discussion of these results. TL-P, JR-M, AG-P, ZG-O, KC-C, IF-M, and JF-T significantly contributed to drafting and critically reviewing the paper. All authors have contributed to the work and agree with the presented findings and that the work has not been published before nor is being considered for publication in another journal. All authors approved the final version of the manuscript and assume accountabilities for all aspects of the work.

## Conflict of Interest

ZG-O received consultancy fees from Lysosomal Therapeutics Inc. (LTI), Idorsia, Prevail Therapeutics, Inceptions Sciences (now Ventus), Ono Therapeutics, Denali, Handl Therapeutics, Neuron23, and Deerfield. The remaining authors declare that the research was conducted in the absence of any commercial or financial relationships that could be construed as a potential conflict of interest.

## Publisher's Note

All claims expressed in this article are solely those of the authors and do not necessarily represent those of their affiliated organizations, or those of the publisher, the editors and the reviewers. Any product that may be evaluated in this article, or claim that may be made by its manufacturer, is not guaranteed or endorsed by the publisher.
