# Corrigendum: Clinical and genetic analysis of Costa Rican patients with Parkinson's disease

**DOI:** 10.3389/fneur.2023.1227084

**Published:** 2023-06-15

**Authors:** Gabriel Torrealba-Acosta, Eric Yu, Tanya Lobo-Prada, Javier Ruíz-Martínez, Ana Gorostidi-Pagola, Ziv Gan-Or, Kenneth Carazo-Céspedes, Jean-François Trempe, Ignacio F. Mata, Jaime Fornaguera-Trías

**Affiliations:** ^1^Department of Neurology and Neurosurgery, Baylor College of Medicine, Houston, TX, United States; ^2^Neurosciences Research Center, Universidad de Costa Rica, San José, Costa Rica; ^3^Montreal Neurological Institute and Hospital, McGill University, Montreal, QC, Canada; ^4^Department of Human Genetics, McGill University, Montreal, QC, Canada; ^5^Department of Biochemistry, Medicine School, Universidad de Costa Rica, San José, Costa Rica; ^6^Group of Neurodegenerative Diseases, Biodonostia Health Research Institute, San Sebastian, Spain; ^7^CIBERNED, Centro de Investigación Biomédica en Red sobre Enfermedades Neurodegenerativas, Madrid, Spain; ^8^Movement Disorders Unit, Neurology Department, Donostialdea Integrated Health Organisation, Osakidetza Basque Health Service, San Sebastian, Spain; ^9^Genomic Platform, Biodonostia Health Research Institute, San Sebastian, Spain; ^10^Department of Neurology and Neurosurgery, McGill University, Montreal, QC, Canada; ^11^Department of Neurology, Hospital San Juan de Dios, Caja Costarricense de Seguro Social, San José, Costa Rica; ^12^Department of Pharmacology and Therapeutics and Centre de Recherche en Biologie Structurale, McGill University, Montreal, QC, Canada; ^13^Cleveland Clinic Foundation, Genomic Medicine, Lerner Research Institute, Cleveland, OH, United States

**Keywords:** Parkinson's disease, genotype, phenotype, Costa Rica, Latin America

In the published article, there was an error. The stated number of protocols needed to be corrected. These errors were identified after an audit conducted by the CONIS (Consejo Nacional de Investigacion en Salud) of Costa Rica.

A correction has been made to the Ethics Statement. This statement previously stated:

The studies involving human participants were reviewed and approved by Ethics Committee of Hospital San Juan de Dios, Caja Costarricense de Seguro Social (CLOBI-HSJD #014- 2015) and the University of Costa Rica (837-B5-304). The patients/participants provided their written informed consent to participate in this study.

The corrected statement appears below:

The studies involving human participants were reviewed and approved by the Ethics Committee of the University of Costa Rica (VI-3668-2014 and final report 837-B5-302). The patients/participants provided their written informed consent to participate in this study.

The authors apologize for this error and state that this does not change the scientific conclusions of the article in any way. The original article has been updated.

