# Supplementary material for: Clinical and Genetic Analysis of Costa Rican Patients With Parkinson's Disease
Source: Front Neurol. 2021 Aug 4;12:656342. doi: 10.3389/fneur.2021.656342 (PMC8371686; doi:10.3389/fneur.2021.656342)
Supplement: Supplementary file 1 [file Data_Sheet_1.ZIP › Supplementary Figure 1.docx]

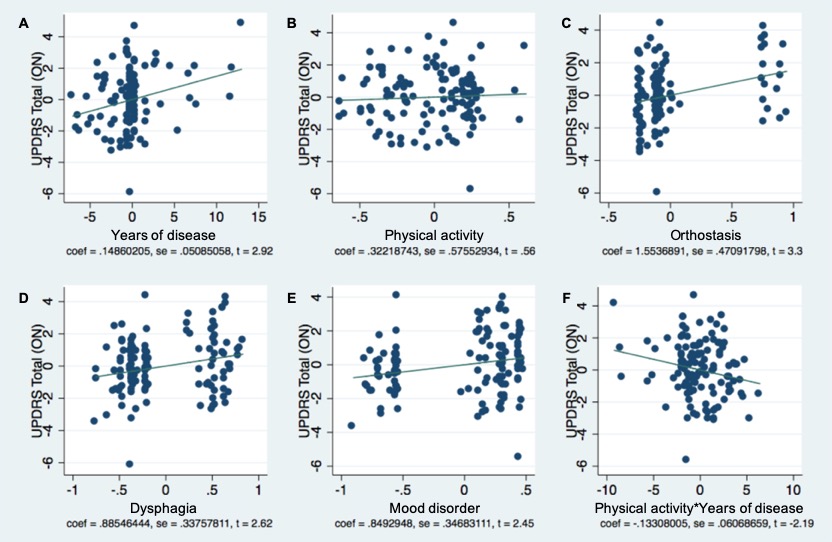


**Supplementary Figure 1.** Multivariate linear regression model coefficients scatter plot assessing the relationship between clinical features of PD and total ON UPDRS score. In this model, increasing years of evolution of the disease (A) along with the presence of orthostasis (C), dysphagia (D) and mood disorders (E) significantly correlated with increased scores in total ON UPDRS. Furthermore, we found an interaction between performing regular physical activity and years of disease (F), where despite having increased years of disease, patients that performed regular physical activity still scored less in the total ON UPDRS.
