# Supplementary material for: Clinical and Genetic Analysis of Costa Rican Patients With Parkinson's Disease
Source: Front Neurol. 2021 Aug 4;12:656342. doi: 10.3389/fneur.2021.656342 (PMC8371686; doi:10.3389/fneur.2021.656342)
Supplement: Supplementary file 1 [file Data_Sheet_1.ZIP › Supplementary Figure 2.docx]

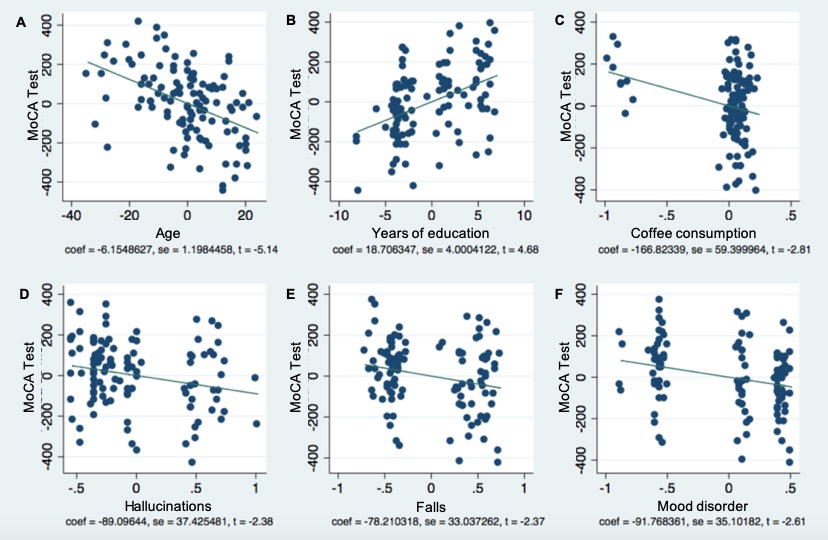


**Supplementary Figure 2.** Multivariate linear regression model coefficients scatter plot for MoCA test scores. In this model, lower scores in MoCA test significantly correlated with increasing age (A), coffee consumption (C) and the presence of hallucinations (D), falls (E) and mood disorders (F), whereas increasing years of education (B) correlated with better MoCA scores.
