# Supplementary material for: Clinical and Genetic Analysis of Costa Rican Patients With Parkinson's Disease
Source: Front Neurol. 2021 Aug 4;12:656342. doi: 10.3389/fneur.2021.656342 (PMC8371686; doi:10.3389/fneur.2021.656342)
Supplement: Supplementary file 1 [file Data_Sheet_1.ZIP › Supplementary Table 1.docx]

**Supplementary Table 1.** Average coverage of the 10 genes analyzed in this study along with the percentage of nucleotides covered at >15X and >30X for each gene.

| Gene | Average coverage | Percentage of nucleotide passing >15x | Percentage of nucleotide passing >30x |
| --- | --- | --- | --- |
| *DJ-1* | 829 | 100 | 100 |
| *ATP13A2* | 178 | 96 | 96 |
| *PINK1* | 671 | 97 | 97 |
| *GBA* | 758 | 100 | 97 |
| *SNCA* | 542 | 98 | 98 |
| *PARK2* | 799 | 98 | 98 |
| *LRRK2* | 470 | 97 | 97 |
| *GCH1* | 554 | 100 | 100 |
| *VPS13C* | 581 | 98 | 96 |
| *VPS35* | 502 | 96 | 96 |
